# Evaluation of the Educational Value of YouTube Videos About Physical Examination of the Cardiovascular and Respiratory Systems

**DOI:** 10.2196/jmir.2728

**Published:** 2013-11-13

**Authors:** Samy A Azer, Hala A AlGrain, Rana A AlKhelaif, Sarah M AlEshaiwi

**Affiliations:** ^1^Curriculum Development and Research UnitDepartment of Medical EducationKing Saud UniversityRiyadh 11461Saudi Arabia

**Keywords:** YouTube, learning resources, medical education, cardiovascular system physical examination, respiratory system physical examination, medical curriculum, clinical skills, self-directed learning, competency

## Abstract

**Background:**

A number of studies have evaluated the educational contents of videos on YouTube. However, little analysis has been done on videos about physical examination.

**Objective:**

This study aimed to analyze YouTube videos about physical examination of the cardiovascular and respiratory systems. It was hypothesized that the educational standards of videos on YouTube would vary significantly.

**Methods:**

During the period from November 2, 2011 to December 2, 2011, YouTube was searched by three assessors for videos covering the clinical examination of the cardiovascular and respiratory systems. For each video, the following information was collected: title, authors, duration, number of viewers, and total number of days on YouTube. Using criteria comprising content, technical authority, and pedagogy parameters, videos were rated independently by three assessors and grouped into educationally useful and non-useful videos.

**Results:**

A total of 1920 videos were screened. Only relevant videos covering the examination of adults in the English language were identified (n=56). Of these, 20 were found to be relevant to cardiovascular examinations and 36 to respiratory examinations. Further analysis revealed that 9 provided useful information on cardiovascular examinations and 7 on respiratory examinations: scoring mean 14.9 (SD 0.33) and mean 15.0 (SD 0.00), respectively. The other videos, 11 covering cardiovascular and 29 on respiratory examinations, were not useful educationally, scoring mean 11.1 (SD 1.08) and mean 11.2 (SD 1.29), respectively. The differences between these two categories were significant (*P*<.001 for both body systems). The concordance between the assessors on applying the criteria was 0.89, with a kappa score >.86.

**Conclusions:**

A small number of videos about physical examination of the cardiovascular and respiratory systems were identified as educationally useful; these videos can be used by medical students for independent learning and by clinical teachers as learning resources. The scoring system utilized by this study is simple, easy to apply, and could be used by other researchers on similar topics.

## Introduction

Current medical curricula are paying increasing attention to learning how to conduct physical examinations and to early exposure of students to clinical skills. The aim is to highlight physical examination as a core competency for students and to ensure that the content and delivery of this component in the curriculum has been mastered before graduation [1,2]. Learning about physical examination of the cardiovascular and respiratory systems, as is the case with other elements of clinical skills, cannot be achieved from reading textbooks or attending lectures. Such skills are usually developed through observation of clinicians performing examinations and through practice on simulated and real patients [3-5].

For many years, students relied on their clinical teachers as one of the main sources for learning such skills [3,4]. However, with the introduction of problem-based learning (PBL) and self-directed learning into most medical curricula, more emphasis has been placed on changing the learning and teaching pedagogy [5-7]. In these courses, students use a range of learning resources, including review papers, journal articles, textbooks, museum specimens, simulated patients, computer-aided learning programs, and multimedia [8-10]. The Internet has become an easily available resource of up-to-date information worldwide [9,11-15]. Medical students usually rely on Google and YouTube as the first resources in their research [12-14].

YouTube is the largest Internet video-sharing site and is a useful tool in social communication, business, advertising, and news as well as a promising learning resource for students and the general public [16,17]. In 2005, YouTube was created as an arena for personal/social communication and for distribution of commercial content. Although similar video-sharing sites are available for public use, YouTube has become the most popular worldwide. Statistical data offered by YouTube provide evidence of its popularity [17]. For example, YouTube is the largest video site with over 4 billion videos watched around the globe every day and one hour of video is uploaded to YouTube every second [17].

Recently, the quality of YouTube videos has been evaluated in a number of areas related to medical and patient health information and medical skills [18], including first aid information on thermal burns [19], human papilloma virus vaccinations [20], investigation into the mechanisms of elbow dislocation [21], clinical procedures [22], rheumatoid arthritis [23], as a learning resource for nurses [24], surface anatomy [25], cardiopulmonary resuscitation [26], dental education [27], and infantile spasms [28]. However, not all researchers found YouTube videos to be educationally useful videos; on the contrary, a number of researchers warned that there are thousands of videos on YouTube that promote misleading information and could possibly endanger some viewers. For example, there are videos encouraging anorexia as a healthy life-style choice [29] and non-suicidal self-injury videos that may foster normalization of non-suicidal self-injury and potentially reinforce the behavior through regular viewing [30,31].

With these limitations in mind, there is no doubt that educationally designed videos have the advantage of explaining difficult concepts through using simulation, graphic diagrams, dynamic illustrations, analogies, and simulated patients. The learning/teaching benefits of videos will be enhanced if videos are well-designed, explore scientifically correct content, feature a clear presentation, and address students’ learning needs. Searching for educationally useful videos on YouTube may be time consuming and requires knowledge from researchers about what makes an educationally useful video [32-35].

No studies assessing the usefulness of YouTube videos on examination of the cardiovascular system (CVS) and the respiratory system (RS) could be found in PubMed. This study is aimed at assessing YouTube videos regarding physical examination of the cardiovascular and respiratory systems.

## Methods

### Searching YouTube

From November 2, 2011 to December 2, 2011, the YouTube site was searched using the following key words: “cardiovascular system examination”, “cardiovascular clinical examination”, “cardiovascular physical examination”, “respiratory system examination”, “respiratory clinical examination”, and “respiratory physical examination”. When searching YouTube, quotation marks were used with these terms to specify that these terms must be present. Only the first 10 pages of results for each search term were screened for related videos. The reason for not searching videos beyond page 10 of the search term results is that one is less likely to find videos covering the key words and users are less likely to go that far in their search [26]. We observed that videos not related to search words could be seen by the third and the fourth pages. By pages 8 to 10, videos not related to the search were found. Therefore, it was decided to go only as far as the first 10 pages for each search. Videos in the English language only were identified and the related URL was recorded.

The search was conducted by three authors (HAA, RAA, and SMA) independently using the search key words. The search results were evaluated and used to compile a common pool that was used in further analysis. The inclusion criteria were videos covering clinical examination of the cardiovascular or respiratory systems in adults. Videos were excluded if they were (1) not in the English language, (2) in the form of a lecture, (3) an advertisement or news, (4) discussing signs or symptoms of diseases affecting the cardiovascular or respiratory systems, (5) about simulated patients reflecting on their experiences or roles, (6) about patients with cardiovascular or respiratory disorders, (7) about drugs used in treating patients with cardiovascular or respiratory disorders, or (8) in the form of seminars or reviews of cardiovascular or respiratory systems. Duplicated videos were also excluded and repeats were treated as a single file for analysis. The repeat file with the greatest number of hits was used for analysis.

### Collected Data

For each video, the following data were collected: title, duration of the video, number of days on YouTube, total number of viewers, and name of uploader/creator (organization, group of people, one person). The number of “likes” and “dislikes” (a crude scoring system that viewers can use to assign to each video they watched, though it is not necessarily used by all viewers) for each video was recorded. Because the number of days on YouTube varies widely among videos, we decided to calculate viewership/day as a more accurate parameter compared to total number of viewers. The viewership per day is the ratio of number of viewers to the number of days a video is on YouTube. The number of days is calculated from the day of uploading on YouTube up to December 1, 2011. This calculation of viewership/day was conducted for each video. The page number on which a video was placed was recorded. This is because the YouTube search algorithm is designed to show videos according to their relevance to key words used in the search and hence videos on the first three pages are more likely to be watched than videos on lower pages. Thus, the location of the videos may indirectly affect the number of “likes” and “dislikes” given by viewers.

### Analysis of Videos

The criteria used in evaluating the videos have been described in detail in an earlier work [25,35] with some modification to suit the study. In summary, the design of the criteria is based on four main domains: video content, technical aspects, authority/creator, and pedagogy used. The items in the criteria are grouped under two categories: major and minor. The major criteria comprise: (1) the video uses simulated patients or patients to demonstrate the examination, (2) contents about clinical examination are scientifically correct, (3) images are clear, (4) the creator and/or organization providing the video are mentioned, and (5) sounds are clear and background is free from noise. The minor criteria comprise: (1) the video covers the topic identified in the title, (2) designed at the level of undergraduate medical science students, (3) time to download is reasonable (about 10-15 minutes at the maximum, not interrupted or challenging to download as reported by the three evaluators), (4) information about the creator is up-to-date, (5) the educational objectives are stated, and (6) the topic is clearly presented. The criteria were used to categorize videos into educationally useful and non-educationally useful videos. We mean by “educationally useful” that a video provides scientifically correct and up-to-date knowledge and clinical instructions/skills accepted by educators in other teaching hospitals about cardiovascular and respiratory examinations. As per the basis of the evaluation criteria, educationally useful videos should fulfill the four domains (scientific content, technical aspects, authority/creator, and pedagogy used). Two scores were allocated for each item in the major criteria and one score was allocated to each item under the minor criteria. If an item was fulfilled, an allocated score was given; if not fulfilled, a zero was given. No half scores were used. As per our previous research work, educationally useful videos should fulfill all major criteria items as the minimum requirements plus at least three items under the minor criteria [35].

### Testing the Criteria

To standardize the evaluation of the content of each video and the process of clinical examination, the assessors used the textbook and video by Talley and O’Connor, “Clinical Examination”, as a reference to guide their assessment [36]. The content element in the criteria comprised the following: examiner introduces him/herself to the patient; patient is correctly positioned in bed; examination is conducted within time frame; examination covers the sequence of inspection, palpation, percussion, and auscultation; physical signs are correctly elicited; and examination is conducted in a professional manner. Prior to applying the criteria, we piloted its use. A total of 25 videos were randomly selected and used for this purpose. The criteria were applied independently by three assessors (SMA, HAA, and RAA). None of the assessors shared their findings or discussed the outcome of their evaluation. An Excel spreadsheet covering the three evaluations was examined by a fourth researcher (SAA). The agreement among the assessors was in the range of 96-98%. The findings were discussed among the researchers. The criteria were tested again independently by three assessors for another 25 videos. Videos were then rated independently by three assessors (SMA, HAA, and RAA). When videos were difficult to classify or when there was a disagreement among assessors, all researchers reviewed such videos in a meeting and reached a final agreement. This study was approved by the ethics committee at King Saud University College of Medicine.

### Data Analysis

The data were entered into Microsoft Excel 2010 and were checked before conducting any analysis. Analysis was conducted with SPSS software (version 18.0 for MS Windows) and was reported via mean, SD, percentage, and minimum and maximum; *t* tests and ANOVA (analysis of variance) were performed to determine significant differences. To assess the degree to which different judges or raters agreed in their assessment decisions, Cohen’s kappa for inter-rater reliability was used [37]. Pearson’s correlation coefficient (*r*) was calculated to determine if the viewership per day was correlated to the total scores given to each video. This relationship has also been examined to see if there is a correlation between the number of “likes” given by viewers to a video and the total scores given [38,39]. For all calculations, a *P* value <.05 was considered significant.

## Results

### Videos on CVS and RS Examinations

**Figure 1:**
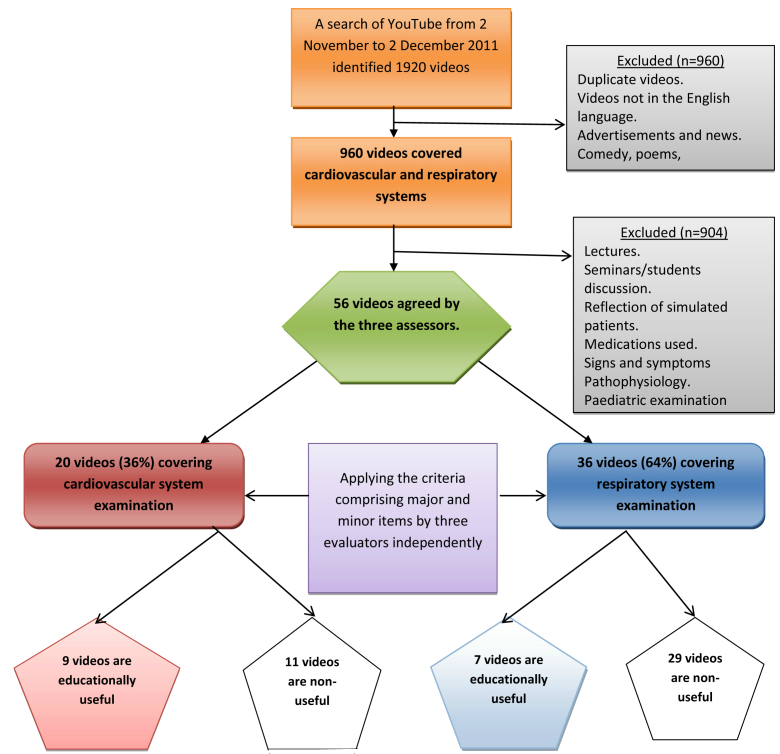
Searching YouTube for videos covering examination of the cardiovascular and respiratory systems.

**Figure 2:**
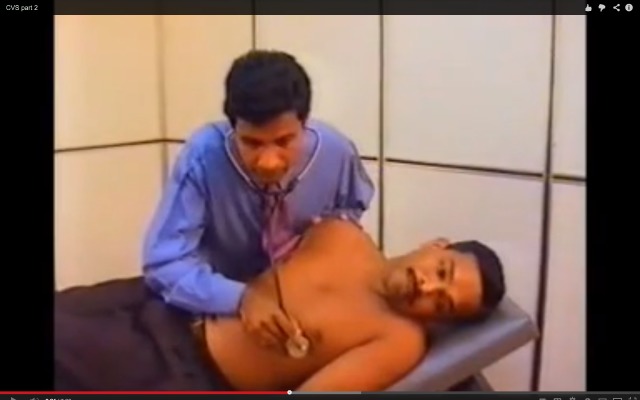
Screenshot of video.

**Figure 3:**
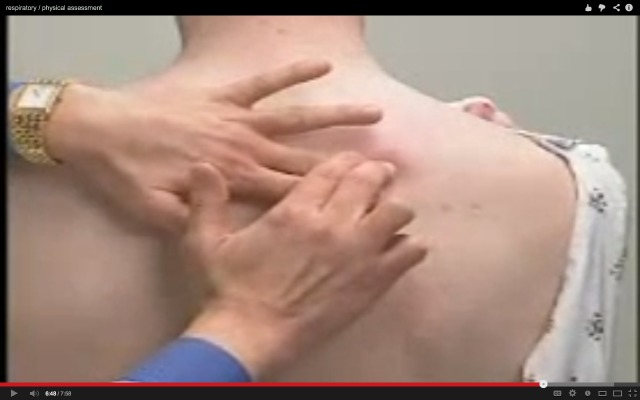
Screenshot of video.

**Figure 4:**
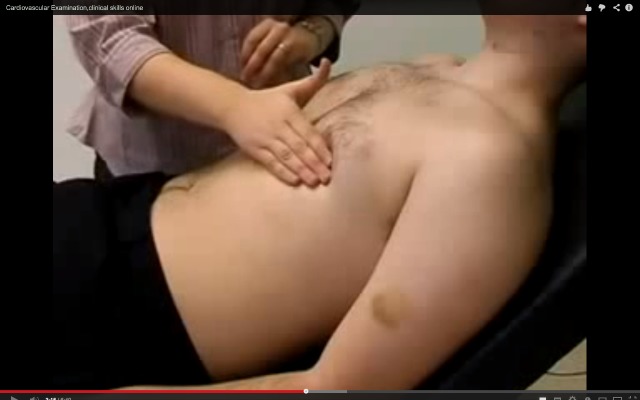
Screenshot of video.

A total of 1920 YouTube videos were found on initial search and, on applying the inclusion criteria and visual examination of the videos, only 56 videos were relevant to clinical examination of the CVS (20 videos) and the RS (36 videos) (Table 1). Figure 1 summarizes how the YouTube searches and the number of videos in each category were refined on the basis of the inclusion and exclusion criteria. Examples of screenshots of these videos are shown in Figures 2-4.

**Table 1:** YouTube videos covering examination of the cardiovascular and respiratory systems (N=56).

Body system		Number of videos	Duration	Total number of days on YouTube	Total viewership	Total scores
		n (%)	minutes (seconds)	n (mean, minimum, maximum)	n (%)	mean (SD)
**Cardiovascular system**						
	Educationally useful	9 (45)	71 (18)	3571 (3968; 20; 1020)	129,350 (29)	14.9 (0.33)
	Educationally not useful	11 (55)	73 (30)	5773 (5248; 154; 955)	315,420 (71)	11.1 (1.08)
	*P* value					<.001
**Respiratory system**						
	Educationally useful	7 (19)	56 (48)	5515 (7879; 219; 1673)	413,483 (41)	15.0 (0.00)
	Educationally not useful	29 (81)	147 (31)	20592 (7101; 31; 1381)	600,183 (59)	11.2 (1.29)
	*P* value					<.001

### Analysis of Videos

The total duration of these video clips was 349 minutes, 11 seconds. The use of the criteria for categorizing the videos into useful and non-useful videos revealed that there were 9 CVS and 7 RS videos that provided useful information on clinical examination. These videos scored mean 14.9 (SD 0.33) and mean 15.0 (SD 0.00), respectively. The total duration of useful videos was 128 minutes, 6 seconds (71 minutes, 18 seconds for the CVS videos and 56 minutes, 48 seconds for the RS videos). The total viewers of all videos were 1,458,436. For both body systems, useful videos were viewed by 542,833 viewers (37.22% of total viewers, 542,833/1,458,436). Table 2 reveals detailed information about the 56 videos included in the study and the multimedia links show examples of useful videos about examination of the cardiovascular system (Multimedia Appendix 1) and the respiratory system (Multimedia Appendix 2).

Useful videos were created by doctors or professional bodies; a link to an organization such as ImedrxTV, geekymedics123, MedicalStudentApps, mosaicism105, or the name of the creator and his or her credentials, such as Professor S. Femando from the Open University of Sri Lanka, were shown. Some videos were linked to universities and known teaching institutes such as Manchester Medical School, St. George’s University of London, Central Manchester University Hospitals, the National Health Service, and the University of Wisconsin School of Medicine and Public Health.

Non-educationally useful videos scored mean 11.1 (SD 1.08) for CVS examination and mean 11.2 (SD 1.29) for the RS examination. Compared to educationally useful videos, the differences were significant (*P*<.001 for both CVS and RS videos) (Table 1). For both body systems, non-useful videos were viewed by 915,603 (62.78% of total viewers, 915,603/1,458,436). Non-educationally useful videos failed due to a number of reasons. The majority of the non-educationally useful videos failed to fulfill one of the major criterion items. Among those, more than 80% (33/40) were due to the image lacking clarity or no mention of the creator of the video. The concordance between the assessors on applying the criteria was 0.89, with a kappa score >.86.

The correlations between the total video scores and the number of viewers/day were not significant for the CVS videos (*r*=.06, *P*=.059) and the RS videos (*r*=.027, *P*=.827). Similarly, no correlation was found between likes or dislikes and the total number of scores given to CVS videos (*r*=.06, *P*=.059) or RS videos (*r*=.02, *P*=.832). No significant correlation was found between the video location on the first pages and the scores for like or dislike.

**Table 2:** Details about the 56 videos identified in the study.

No.	Title	URL	Days on YouTube	Viewership/day	Total score
1^a^	Cardiovascular system	http://www.youtube.com/watch?v=v12EAuyxs6w	721	27.56	15
2	Macleod’s Clinical Examination, Cardiovascular System	http://www.youtube.com/watch?v=8h8h7ee9D84	219	21.84	15
3	Cardiovascular Examination, clinical skills online	http://www.youtube.com/watch?v=E6EOCY-OszE	894	37.68	15
4	Cardiovascular Examination - OSCE guide	http://www.youtube.com/watch?v=SJ3UwKkLyy0	266	69.80	15
5	Cardiovascular Examination	http://www.youtube.com/watch?v=XiM2fnVDg9A	367	111.06	15
6	Cardiovascular Exam	http://www.youtube.com/watch?v=69LuXu42Ahc	1020	9.44	14
7	Cardiovascular Examination	http://www.youtube.com/watch?v=iiPvQcyEfEY	44	43.75	15
8	CVS Examination Part 1	http://www.youtube.com/watch?v=CwM8viJ4M6Q&feature=mfu_in_order&list=UL	20	4.05	15
9	CVS Examination Part 2	http://www.youtube.com/watch?v=MQLJv-1qP_s	20	2.85	15
10^b^	Respiratory System	http://www.youtube.com/watch?v=vD7b-Ery014	721	9.48	15
11	Respiratory System Examination	http://www.youtube.com/watch?v=YK82HlDrvWw	665	18.59	15
12	Respiratory Examination	http://www.youtube.com/watch?v=hWGzi5h2UR8	1673	172.05	15
13	Respiratory / physical assessment	http://www.youtube.com/watch?v=IepL5u5lAtE	1183	55.09	15
14	Macleod’s Clinical Examination, Examination of the Respiratory System	http://www.youtube.com/watch?v=akr40RXu_H8	219	36.27	15
15	04a.Physical Exam –Thorax - part 1/2	http://www.youtube.com/watch?v=axjRJtXc-Xc	527	31.54	15
16	04a.Physical Exam –Thorax - part 2/2	http://www.youtube.com/watch?v=j0lKzPyM7_k	527	31.73	15
17^c^	Physical Assessment of Thorax Lungs and Cardiovascular	http://www.youtube.com/watch?v=cKMROjEaowc	405	12.01	10
18	CVS examination.wmv	http://www.youtube.com/watch?v=C7c-K6Jltaw	294	3.31	12
19	Cardiovascular Examination	http://www.youtube.com/watch?v=hXU24g95wJU	448	183.23	11
20	Cardiovascular Physical.mpg	http://www.youtube.com/watch?v=McOAJxQWb5A	717	1.91	10
21	The One Minute Cardiac Examination	http://www.youtube.com/watch?v=EwL43OwwPYY	569	18.631	13
22	Cardiovascular Examination	http://www.youtube.com/watch?v=BYZJN3Zlllg	666	58.89	10
23	Cardiovascular Examination	http://www.youtube.com/watch?v=-8Hi1PjZam4	903	79.76	12
24	Jugular Venous Pressure (JVP)	http://www.youtube.com/watch?v=4YBXaWWG3Ns	431	55.14	12
25	Inspection Of Chest	http://www.youtube.com/watch?v=SkWrl7t2CE8	154	2.01	12
26	Examination: General Cardiovascular	http://www.youtube.com/watch?v=XRJa7Vina10	231	0.94	11
27	Cardiovascular examination	http://www.youtube.com/watch?v=U-fyQfjL9aQ	955	83.75	10
28^d^	Examination of Respiratory System Part 1 General Exam	http://www.youtube.com/watch?v=4xPxX94XANo	81	6.15	11
29	04b.Anterior and posterior Thorax and Axilla	http://www.youtube.com/watch?v=_xt3nXnEoqY	528	214.60	12
30	Respiratory Physical.mpg	http://www.youtube.com/watch?v=hkxH0fycRVE	717	6.79	13
31	Clinical Skills Session – Resp. Exam	http://www.youtube.com/watch?v=uRg7zg1wCec	1381	154.79	11
32	9-Thorax and Lungs-Examining the Anterior Thorax and Lungs	http://www.youtube.com/watch?v=bYpJ0DYtxnE	162	2.03	11
33	Respiratory Examination	http://www.youtube.com/watch?v=a-4BUharWMA	666	51.55	12
34	HOW Examination of Respiratory System Part 1 of 2	http://www.youtube.com/watch?v=OX1Y21K41rs	67	1.73	10
35	HOW Examination of Respiratory System Part 2 of 2	http://www.youtube.com/watch?v=ol44OFQMHz0	67	1.97	10
36	Medical Gallery - Loyola Full Thorax Exam Part 1	http://www.youtube.com/watch?v=hTagtppnCIk	468	1.82	12
37	Medical Gallery - Loyola Full Thorax Exam Part 2	http://www.youtube.com/watch?v=TvLX0EP7fc0	468	0.30	11
38	Deb Video 6 - Respiratory Exam.mpg	http://www.youtube.com/watch?v=ud6rV3kVdjI&feature=mfu_in_order&list=UL	722	10.77	10
39	Deb Video 7 - Respiratory Exam.mpg	http://www.youtube.com/watch?v=WLt5kY15BBE	722	16.47	8
40	Respiratory Mohamed	http://www.youtube.com/watch?v=VBTrAz_LwJE	397	0.78	11
41	[Respiratory Mohamed] Part 2	http://www.youtube.com/watch?v=QcTAsQew8Cc	397	0.75	11
42	[Respiratory Mohamed] Part 3	http://www.youtube.com/watch?v=6WtQqfSs9D0	31	25.42	11
43	[Respiratory Mohamed] Part 4	http://www.youtube.com/watch?v=M2bmOrL_ouI	31	9.19	11
44	[Respiratory Mohamed] Part 5	http://www.youtube.com/watch?v=PY8pA0cB2ko	31	9.29	11
45	Examination of the thoracic and respiratory system 1	http://www.youtube.com/watch?v=3emIM6BsWdw	1295	18.25	13
46	Examination of the thoracic and respiratory system 2	http://www.youtube.com/watch?v=vez8Vzm70kc	1295	12.84	13
47	Examination of the thoracic and respiratory system 3	http://www.youtube.com/watch?v=ygP03M0yIwE	1295	27.10	13
48	Examination of the thoracic and respiratory system 4	http://www.youtube.com/watch?v=ABuAMGHrBVM	1295	12.95	11
49	Examination of the thoracic and respiratory system 5	http://www.youtube.com/watch?v=ifnqBzmidDY	1295	7.85	11
50	Examination of the thoracic and respiratory system 6	http://www.youtube.com/watch?v=Us5M1gCxBC8	1295	4.96	11
51	Examination of the thoracic and respiratory system 7	http://www.youtube.com/watch?v=S9ew7uZipbA	1295	7.78	11
52	Examination of the thoracic and respiratory system 8	http://www.youtube.com/watch?v=saWuYMkXQGM	1295	43.14	11
53	Examination of the thoracic and respiratory system 9	http://www.youtube.com/watch?v=GNcmPcLZHi4	1295	7.48	11
54	Examination of the thoracic and respiratory system 10	http://www.youtube.com/watch?v=TkA0CqLuYfM	1295	7.70	11
55	Respiratory Examination (part 1) for OSCEs	http://www.youtube.com/watch?v=OdkdJZ_ppOI	353	24.81	10
56	Respiratory Examination (part 2) for OSCEs	http://www.youtube.com/watch?v=OYDeI2HEyZw&feature=related	353	20.20	10

^a^From 1 to 9 – educationally useful videos on cardiovascular examination (n=9)

^b^From 10 to 16 – educationally useful videos on respiratory examination (n=7)

^c^From 17 to 27 – non-educationally useful videos on cardiovascular examination (n=11)

^d^From 28 to 56 – non-educationally useful videos on respiratory examination (n=29)

## Discussion

### Principal Findings

The aim of this study was to conduct an analysis of YouTube videos about CVS and RS physical examinations and to categorize these videos for educational purposes. Several methods have been described in evaluating videos [40-43]. However, none of these systems were useful for classifying videos about clinical skills. The system used in this study is simple, easy to apply, and covers four key elements, namely: scientific content, technicality, authority, and pedagogy parameters. It has also been tested in two previous studies and has shown a high level of inter-rater correlation while covering the key elements required for an educationally useful video [25,35].

The findings in this study indicated that there are 9 educationally useful videos on CVS examination and 7 on RS examination. These videos provided approximately 71 minutes, 18 seconds of CVS examination and 56 minutes, 48 seconds of RS examination that can be used in clinical learning and teaching purposes. Useful videos were linked mainly to universities or educational institutes. This indicates the involvement of universities and teaching institutes in promoting the use of educational videos as a resource to learners. This is particularly important with the shift of most universities toward self-directed learning and student-centered programs.

YouTube’s search engine, despite all precautions taken to target the search results to videos about examination of these two body systems, delivered more than 1800 videos that were not related to the search terms. Furthermore, the lack of correlation between the total score given to a video and the location of the video when searched (the page number) highlights the fact that the YouTube search algorithm is not well calibrated and unrelated videos usually appear despite the search filter provided by YouTube. Also, the lack of correlation between the total score given to a video to the “like” and “dislike” numbers as well as to viewership/day suggests the possibility that many viewers were watching substandard videos in their learning. Assuming that the majority of these viewers were medical/health students and trainees, these results highlight the need for YouTube to improve its search algorithm system to generate more accurate lists of videos that match with the search terms.

Much emphasis has been placed on physical examination in current medical curricula. To learn such skills, students usually rely on observing clinicians conducting examinations and then practicing on simulated patients and real patients. Recently, Duvivier et al (2012) in a study from Maastricht University reported that on average students devote 20% of their self-study time to skills training on physical examination [3]. They found that students use textbooks, examination guidelines, scientific articles, the Internet, videos/DVD, and scoring forms from previous OSCEs as their learning resources. Although the Internet and videos were among these resources, the study did not explore the sources of these videos and whether YouTube videos were used. Also, the study did not specify how much time they spend on average on resources such as videos and the Internet [3].

There is no substitute for witnessing a physical examination or a clinical procedure being performed live. Neither static images nor a description in a book outlining these techniques can offer the same impact as personal experience. Videos, however, are a medium that can transfer the experience and help in mastering such skills through repeated watching of techniques used and information provided. Online videos have become a routine and important tool in a student’s preparation for clinical skills. Attending clinical skills sessions, reading textbooks along with watching online videos, and practicing skills learned on patients have become important learning strategies in most clinical schools [44,45]. Based on cognitive psychological research, the use of videos will help expose students to the techniques of clinical examination, approaches for examining patients, and how to manage the sequence of technical steps in such examinations [45-50].

Considering the increasing number of learners using the Internet as their primary source of information, medical educators and clinical teachers should recognize the importance of YouTube in education and invest in using Web 2.0 in learning and teaching activities such as clinical teaching [8,13-14]. Although there are other links on the Web that provide free videos, we decided to examine the videos on YouTube for a number of reasons. First, YouTube is popular and usually preferred by users compared to other websites. Second, it is relatively easy to share videos on YouTube. Third, there is continuous improvement in the design of the YouTube site and each video is accompanied by useful data that reflect the evolution of online social networking and can be of use to researchers and viewers. Finally, YouTube has succeeded in providing social networking and enabling discussion among viewers, which could be useful to viewers as they share useful videos with each other.

This study shows that clinical teachers who are competent in clinical teaching and clinical educators, such as those who created the videos we identified in this study, can offer great service to medical and health students worldwide by placing their work on sharing websites such as YouTube. This can be part of knowledge transfer and scholarly work as it is created by academics, shared, and peer-reviewed [50]. Recently, YouTube launched YouTube EDU, an area of YouTube where video creators must possess high-quality credentials and provide evidence of significant mass of resources before they qualify to have their content included in YouTube EDU. This may have a major impact on improving successful search strategies particularly for YouTube videos designed for educational purposes.

### Limitations

This study represents a snapshot of available resources during November/December 2011, and, since then, there may have been more videos uploaded and made available. Given the continuous upload of videos on YouTube and the volume of new videos added to the system on a daily basis, further studies are needed to assess whether new videos of high quality and coverage have been added.

The small number of videos used in this study is a limitation to this study. The study was limited to videos in the English language and only those covering examination of adults, which may have contributed to the smaller number of videos found. However, the majority of videos uploaded on YouTube are in the English language. Also, there is the possibility that some videos were not labeled as such and thus were unidentifiable under our search terms, despite all our precautions including conducting the search of YouTube over 30 days independently by three researchers and using six search keywords to compile a common pool. The search in this study was limited to YouTube and there is the possibility that videos on other websites, such as those of medical and other health professional societies and medical journals, were not included.

### Conclusions

This is possibly the first study assessing the educational value of YouTube videos about physical examination of the CVS and RS systems. Despite the small number of videos identified and found educationally useful, these videos can be used by medical students for independent learning and by clinical teachers as learning resources. The scoring system utilized by this study is simple, easy to apply, and could be used by other researchers on similar topics. The authors encourage other researchers to assess the tool and contribute to its improvement.

## Multimedia Appendix 1

Useful cardiovascular video.

## Multimedia Appendix 2

Useful respiratory video.

## Abbreviations

CVS: cardiovascular system
PBL: problem-based learning
RS: respiratory system
